# Cost-effectiveness analysis of trifluridine/tipiracil combined with bevacizumab vs. monotherapy for third-line treatment of colorectal cancer

**DOI:** 10.3389/fpubh.2024.1465898

**Published:** 2024-11-13

**Authors:** Long-Zhuan Huang, Ya-Qing Chen, Hang-Ye Gu, Yong Chen

**Affiliations:** Key Specialty of Clinical Pharmacy, The First Affiliated Hospital of Guangdong Pharmaceutical University, Guangzhou, China

**Keywords:** trifluridine/tipiracil, bevacizumab, colorectal cancer, cost-effectiveness analysis, Markov model

## Abstract

**Background:**

The combination of trifluridine/tipiracil (FTD/TPI) and bevacizumab has demonstrated promising efficacy and safety in the treatment of colorectal cancer (CRC). This study aims to evaluate the cost-effectiveness of trifluridine/tipiracil combined with bevacizumab vs. trifluridine/tipiracil monotherapy as a third-line treatment regimen for colorectal cancer within the Chinese healthcare system, providing an economic basis for clinical application.

**Methods:**

Based on data from the SUNLIGHT Phase III clinical trial, a dynamic Markov model was constructed with a cycle length of 4 weeks and a simulation duration of 10 years. Direct medical costs and quality-adjusted life years (QALYs) were calculated. The incremental cost-effectiveness ratio (ICER) was compared with the willingness-to-pay threshold (WTP = ¥268,200.00/QALY) to assess the economic viability of the treatment regimen. One-way sensitivity analysis and probabilistic sensitivity analysis were conducted to verify the robustness of the model results.

**Results:**

The cost of trifluridine/tipiracil combined with bevacizumab treatment (¥838,492.74) was higher than that of trifluridine/tipiracil monotherapy (¥357,396.97), with greater health benefits (2.45 QALYs vs. 1.54 QALYs). The ICER was ¥527,577.36/QALY, exceeding the willingness-to-pay threshold. One-way sensitivity analysis indicated that drug costs and utility values during the progression-free period significantly impacted model outputs. Probabilistic sensitivity analysis further confirmed the robustness of the results, showing that at a willingness-to-pay threshold of ¥494,000.00, the probability of the combined treatment being cost-effective was 50%.

**Conclusion:**

Trifluridine/tipiracil combined with bevacizumab, as a third-line treatment for colorectal cancer, does not have a cost-effectiveness advantage compared to trifluridine/tipiracil monotherapy in economic evaluations.

## 1 Introduction

Colorectal cancer refers to malignant epithelial tumors originating from the colon or rectum, encompassing malignancies in the cecum, appendix, ascending colon, hepatic flexure, transverse colon, splenic flexure, descending colon, and rectum (1). Globally, CRC ranks third in incidence and second in mortality, posing a significant public health burden and emerging as a global public health challenge (2–4). In China, the incidence and mortality rates of CRC are on the rise, currently making it the second most common cancer after lung cancer (5, 6).

First-and second-line treatment options for colorectal cancer mainly include fluorouracil-based chemotherapy in combination with oxaliplatin and irinotecan, as well as treatments targeting vascular endothelial growth factor (VEGF) (mainly using bevacizumab) or epidermal growth factor receptor (EGFR) (the latter is primarily indicated for RAS wild-type tumors) (7). According to the recommendations of the European Society for Medical Oncology (ESMO) (8), 2023 guidelines for metastatic colorectal cancer and the 2023 guidelines from the Chinese Society of Clinical Oncology (CSCO) (9) for colorectal cancer diagnosis and treatment, trifluridine/tipiracil combined with bevacizumab is recommended as a first-line treatment option for metastatic CRC (10). This recommendation is based on data from the SUNLIGHT Phase III clinical trial, which demonstrated that the combination therapy showed superior efficacy and safety compared to trifluridine/tipiracil monotherapy, with a significant extension in progression-free survival (PFS) (10.8 months vs. 7.5 months) and an increase in the 12-month overall survival (OS) rate (43% vs. 30%) (11). Given the lack of health economic studies on the combination therapy of trifluridine/tipiracil and bevacizumab in China, this study aims to evaluate the cost-effectiveness of this combination therapy compared to trifluridine/tipiracil monotherapy from the perspective of the Chinese healthcare system. The study is based on the latest efficacy and safety data from the SUNLIGHT clinical trial and real-world data to provide an economic basis for clinical decision-making in treating CRC patients in China.

## 2 Methods

### 2.1 Patient characteristics

The simulated population in this study is consistent with the SUNLIGHT clinical trial participants, who were histologically diagnosed with unresectable colorectal adenocarcinoma, had received up to two prior chemotherapy regimens with disease progression or intolerance to the last regimen, were able to swallow oral tablets, and had an expected survival of ≥12 weeks.

### 2.2 Treatment regimens

#### 2.2.1 Trifluridine/tipiracil combined with bevacizumab group

Patients were administered trifluridine/tipiracil at a dose of 35 mg/m^2^ orally within 1 h after breakfast and dinner, from day 1 to day 5 and day 8 to day 12, with drug holidays on day 6–7 and day 13–14. Each treatment cycle lasted 2 weeks, followed by a 14-day rest period. Bevacizumab was administered at a dose of 5 mg/kg via intravenous infusion every 2 weeks, specifically on day 1 and day 15 of each treatment cycle. This regimen was repeated every 4 weeks.

#### 2.2.2 Trifluridine/tipiracil monotherapy group

Patients were administered trifluridine/tipiracil at a dose of 35 mg/m^2^ orally within 1 h after breakfast and dinner, from day 1 to day 5 and day 8 to day 12, with drug holidays on day 6–7 and day 13–14. Each treatment cycle lasted 2 weeks, followed by a 14-day rest period.

Given that the SUNLIGHT clinical trial did not disclose subsequent treatment regimens and proportions, this study utilized fruquintinib as the subsequent treatment after disease progression, based on the CSCO 2023 guidelines for colorectal cancer diagnosis and treatment.

### 2.3 Model structure

**Figure 1:**
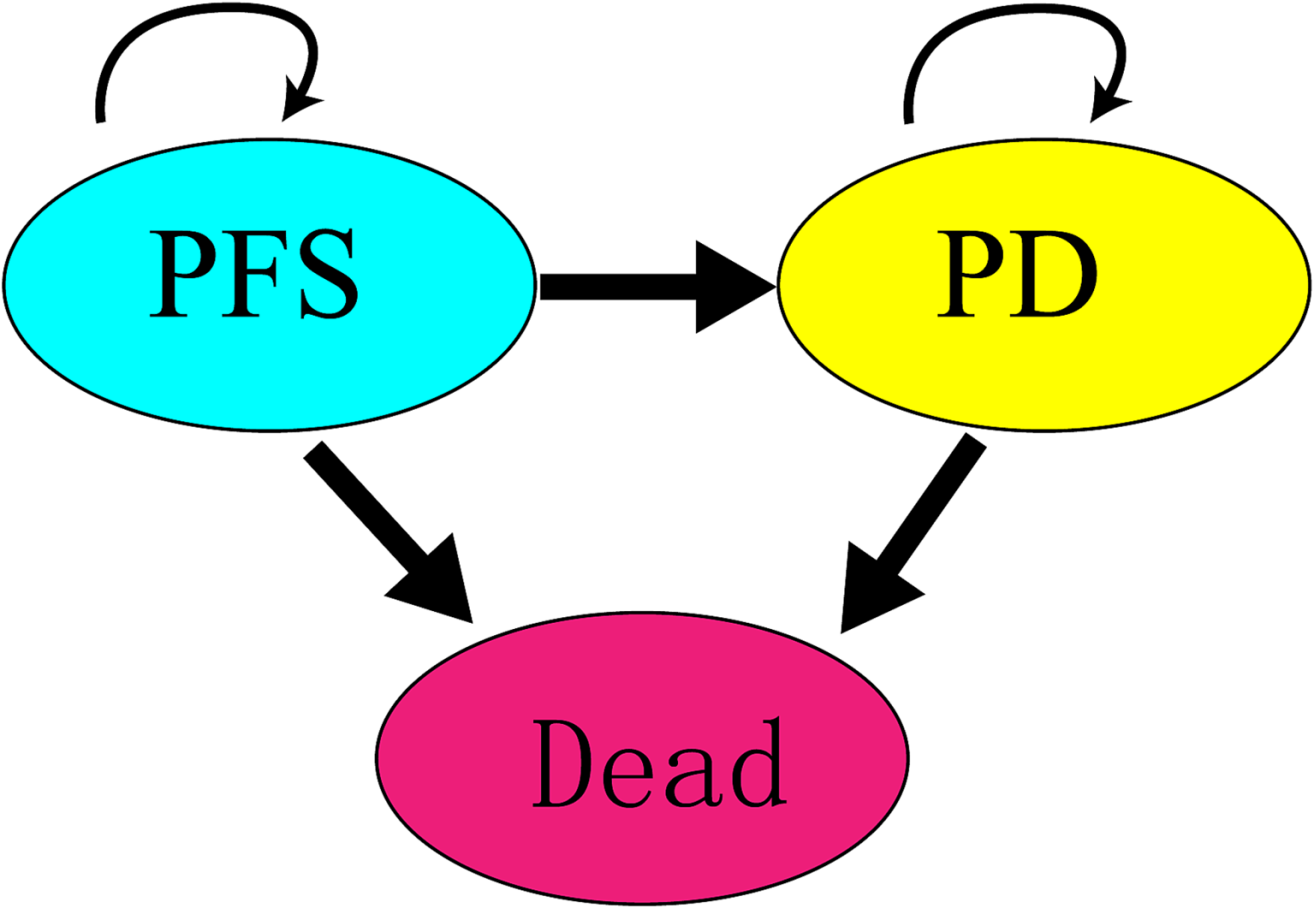
Markov model transition diagram for each state.

A Markov model was constructed using TreeAge Pro 2011 software, incorporating three states: PFS, progressed disease (PD), and death. Initially, all participants were assumed to be in the PFS state. Over time, participants could transition from PFS to PD or death, with transitions being irreversible until all participants reached the death state. The model’s simulation cycle was set to 10 years, with a cycle length of 28 days, considering the administration schedule from the SUNLIGHT clinical trial and key factors such as survival duration and average age of participants. The state transition relationships in the Markov model are illustrated in Figure 1.

According to the “Chinese Guidelines for Pharmacoeconomic Evaluations 2020,” a discount rate of 5% was applied. The willingness-to-pay (WTP) threshold was set at three times the *per capita* GDP of China in 2023. The model’s output indicators included total costs, QALYs, and ICER for the two treatment groups. The ICER and WTP were used for comparison; if the ICER was less than or equal to the threshold, the combination therapy of trifluridine/tipiracil and bevacizumab was considered more cost-effective compared to trifluridine/tipiracil monotherapy. If the ICER exceeded the threshold, trifluridine/tipiracil monotherapy was deemed more cost-effective compared to combination therapy.

### 2.4 Extraction of survival data and calculation of transition probabilities

**Figure 2:**
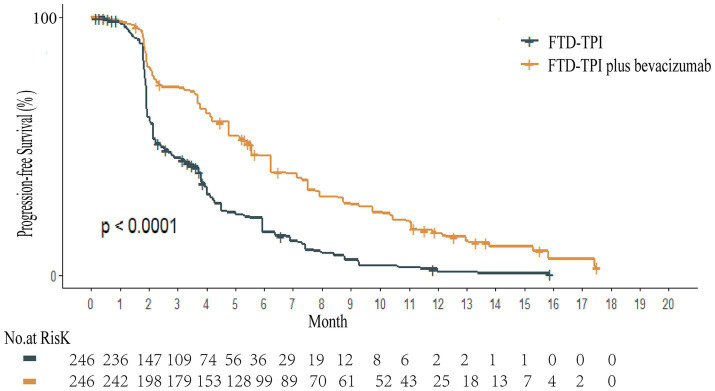
Overall survival reconstruction curve.

**Figure 3:**
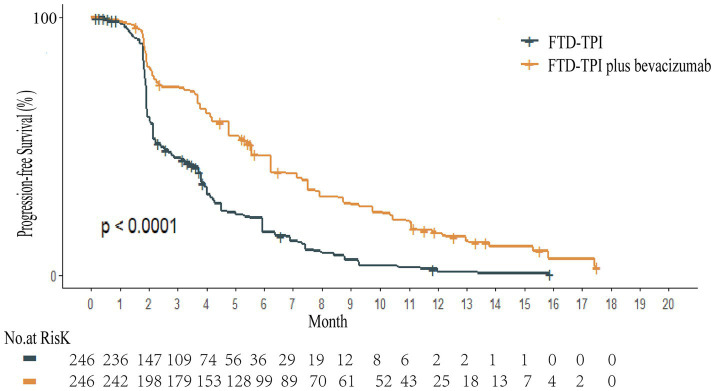
Progression-free survival reconstruction curve.

Kaplan–Meier survival curves for overall survival (OS) and PFS were digitized using the GetData Graph Digitizer software to extract time points and corresponding survival probabilities. The original survival curves were reconstructed using R software, as depicted in Figures 2, 3. Common parametric survival models, including Exponential, Weibull, Log-logistic, Log-normal, Gompertz, and Gamma distributions, were fitted to the data. The goodness of fit was evaluated using the Akaike Information Criterion (AIC) and Bayesian Information Criterion (BIC) (12).

**Figure 4:**
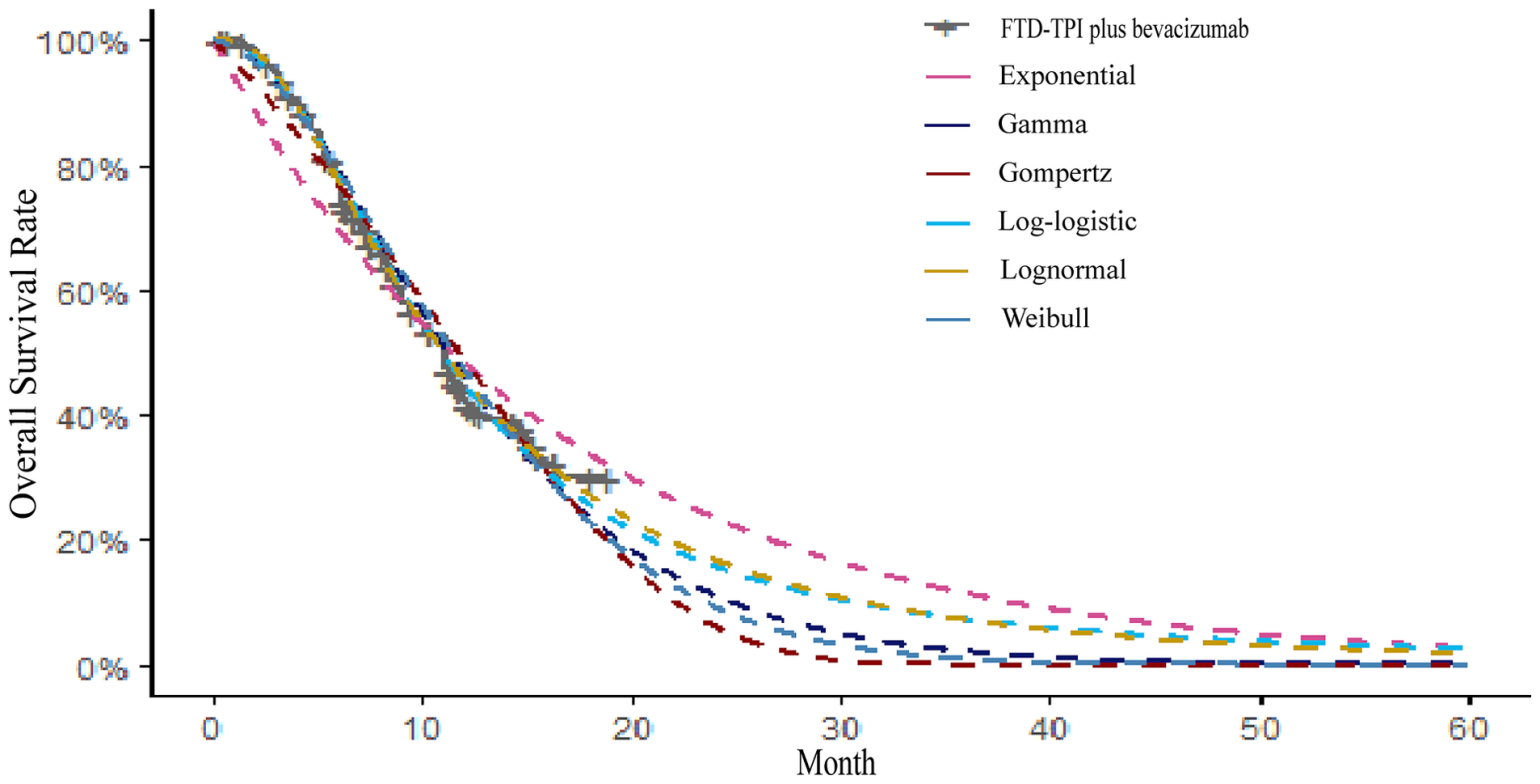
Parameters of OS curve fitting for FTP-TPI plus bevacizumab.

**Figure 5:**
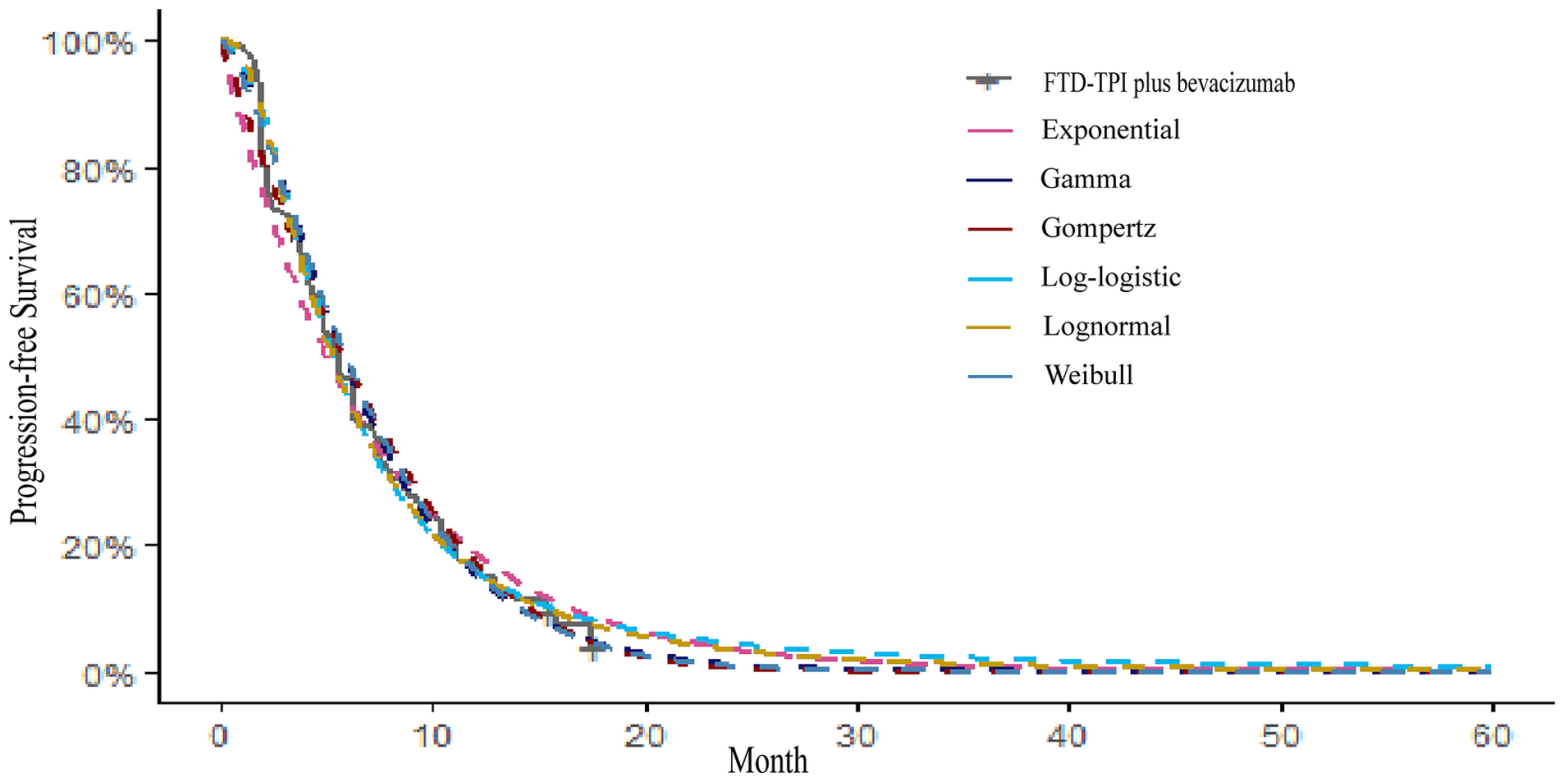
Parameter plot of PFS curve fitting for FTP-TPI plus bevacizumab.

The AIC and BIC results are presented in the Supplementary Table S1, and the fitted curves are shown in Figures 4, 5 (Supplementary Figures S1, S2). Based on the AIC, BIC, and visual inspection results, the Log-normal distribution was identified as the best-fitting model. Therefore, survival curves were extrapolated using the Log-normal distribution, with the shape parameter (*γ*) and scale parameter (*λ*) presented in Table 1. Transition probabilities were calculated using the cumulative survival function S(t) and the cumulative hazard function F(t) of the Log-normal distribution. For the OS curve, the probability of remaining alive (Psts) and the probability of death (Pstd) were calculated as $\mathrm{Pstd}=S\left(t\right)/S\left(t-u\right)$ and Psts=1−Pstd , respectively. Similar calculations were performed for the PFS curve (13–15).

**Table 1:** Shape and scale parameters of the best-fitting model.

Group	Fitting curve	Fitting curve	Shape parameter (*γ*)	Scale parameter (*λ*)
FTP-TPI plus bevacizumab	PFS	Log-normal	1.660	0.850
	OS	Log-normal	2.398	0.808
FTP-TPI	PFS	Log-normal	1.110	0.644
	OS	Log-normal	2.030	0.793

### 2.5 Cost data and health utility values

From the perspective of the Chinese healthcare system, this study only considered direct medical costs, adverse drug event (ADE) treatment costs, follow-up costs for PFS patients, supportive care costs, and end-of-life care costs. ADE treatment costs included only those ADEs with an incidence rate of ≥20% and severity of ≥Grade 3. Drug price data were obtained from the Guangzhou Drug and Medical Consumables Procurement Platform,^1^ while other cost data were sourced from published literature. Dosage calculations were based on the average height (male: 169.70 cm, female: 158.00 cm) and weight (male: 69.60 kg, female: 59.00 kg) (16) of Chinese individuals as reported in 2020, and the gender ratio (256:236) from the SUNLIGHT clinical trial. The average patient height was set at 164.09 cm and average weight at 64.52 kg, resulting in a body surface area (BSA) of 1.79 m^2^( BSA=0.0061×height+0.0124×weight−0.0099 ) (17).

Health state utility values (HSUVs) reflect preferences for specific health states, ranging from 0 to 1. Due to the lack of authoritative utility value studies for Chinese CRC patients, this study referenced similar studies based on Chinese populations (18–21), selecting PFS and PD utility values of 0.84 and 0.57, respectively (22). The costs and parameters are summarized in Table 2.

**Table 2:** Parameters and distributions of the Markov model.

Parameters	Baseline Values	Lower Limits	Upper Limits	Distribution	Sources
Cost of medication per cycle (CNY)					
FTP-TPI plus bevacizumab	23625.40	18900.32	28350.48	Gamma	Procurement platform
FTP-TPI	13947.40	11157.92	16736.88	Gamma	Procurement platform
Follow-up cost (CNY per visit)					
Laboratory tests	317.36	253.89	380.83	Gamma	(18)
Imaging tests	677.70	542.16	813.24	Gamma	(40)
End-of-life care	11299.00	9039.20	13558.80	Gamma	(41)
Supportive treatment	2141.20	1712.96	2569.44	Gamma	(40)
Subsequent treatment cost	7541.1	6032.88	9.49.32	Gamma	Procurement platform
Adverse event management cost (CNY per event)					
Neutropenia	3214.90	2571.92	3857.88	Gamma	(42)
Nausea	298.00	238.40	357.60	Gamma	(43)
Anemia	531.00	424.80	637.20	Gamma	(42)
Fatigue	20.80	16.64	24.96	Gamma	(44)
Tiredness	293.01	234.41	351.61	Gamma	(45)
Diarrhea	276.00	220.80	331.20	Gamma	(46)
Decreased appetite	705.4	564.32	846.48	Gamma	(43)
Utility value					
PFS utility value	0.84	0.67	1.00	Beta	(22)
PD utility value	0.57	0.46	0.68	Beta	(22)
Other parameters					
Discount rate (%)	5	0	8	Beta	
Incidence of adverse events (%)					
FTP-TPI plus bevacizumab group					
Neutropenia	43.10				
Nausea	1.60				
Anemia	6.10				
Fatigue	4.10				
Tiredness	1.20				
Diarrhea	0.80				
Decreased appetite	0.80				
FTP-TPI group					
Neutropenia	32.10				
Nausea	1.60				
Anemia	11.00				
Fatigue	4.10				

The drug prices on the procurement platform as of June 5, 2024, are as follows: Trifluridine and Tipiracil Tablets: ¥6,549.60 [containing Trifluridine 20 mg and Tipiracil Hydrochloride 9.42 mg (equivalent to Tipiracil 8.19 mg), 20 tablets/box, aluminum foil packaging]. Bevacizumab Injection: ¥1,500.00 (4 mL: 0.1 g, 1 vial/box, vial packaging); the incidence rate (%) of adverse reactions is derived from the SUNLIGHT Phase III clinical trial.

### 2.6 Sensitivity analysis

Model stability was verified through one-way sensitivity analysis and probabilistic sensitivity analysis. In the one-way sensitivity analysis, parameters were varied within a reasonable range using 95% confidence intervals (CIs) as the upper and lower limits. If 95% of CIs were unavailable, the baseline value ±20% was used. The discount rate ranged from 0 to 8%. The impact of independent parameter changes on the model was explored, and results were presented as a tornado diagram (23, 24). Probabilistic sensitivity analysis involved 10,000 Monte Carlo simulations based on parameter ranges and probability distributions (beta distribution for health utility values and discount rates, Gamma distribution for cost data). Results were displayed using incremental cost-effectiveness scatter plots and cost-effectiveness acceptability curves.

## 3 Results

### 3.1 Base case analysis

The base case analysis results are shown in Table 3. According to Table 3, the incremental effect of the trifluridine/tipiracil combined with the bevacizumab treatment regimen was 0.91 QALYs, with an incremental cost of ¥481,095.78, resulting in an ICER of ¥527,577.36/QALY. This ICER exceeds the willingness-to-pay threshold of ¥268,200.00, indicating that while the combined treatment regimen increases effectiveness, it also substantially increases costs. Therefore, using a threshold of ¥268,200.00, the trifluridine/tipiracil combined with bevacizumab treatment regimen does not have a cost-effectiveness advantage over trifluridine/tipiracil monotherapy.

**Table 3:** Results of basic analysis.

Group	Cost (CNY)	Incremental cost (CNY)	Effect (QALYs)	Incremental effect (QALYs)	ICER (CNY/QALY)
FTP-TPI plus bevacizumab	838,492.74	481,095.78	2.45	0.91	527,577.36
FTP-TPI	357,396.97		1.54		

### 3.2 Single-factor sensitivity analysis

**Figure 6:**
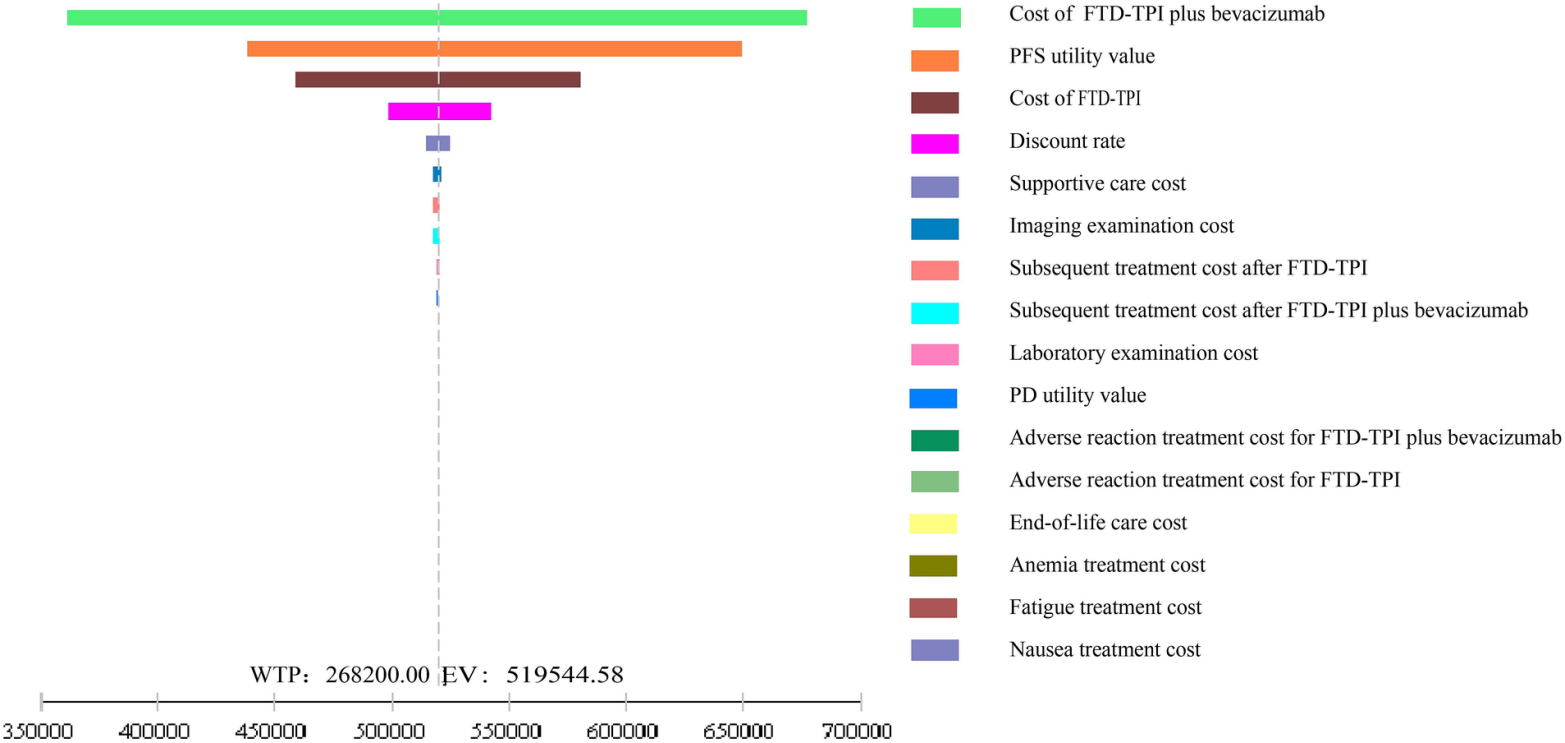
Tornado diagram for one-factor sensitivity analysis.

The results of the one-way sensitivity analysis are shown in Figure 6. The analysis demonstrated that within the reasonable parameter variation range, the model results remained stable, confirming the robustness of the base case analysis. Drug costs, PFS utility values, and discount rates had significant impacts on the model’s output.

### 3.3 Probabilistic sensitivity analysis

**Figure 7:**
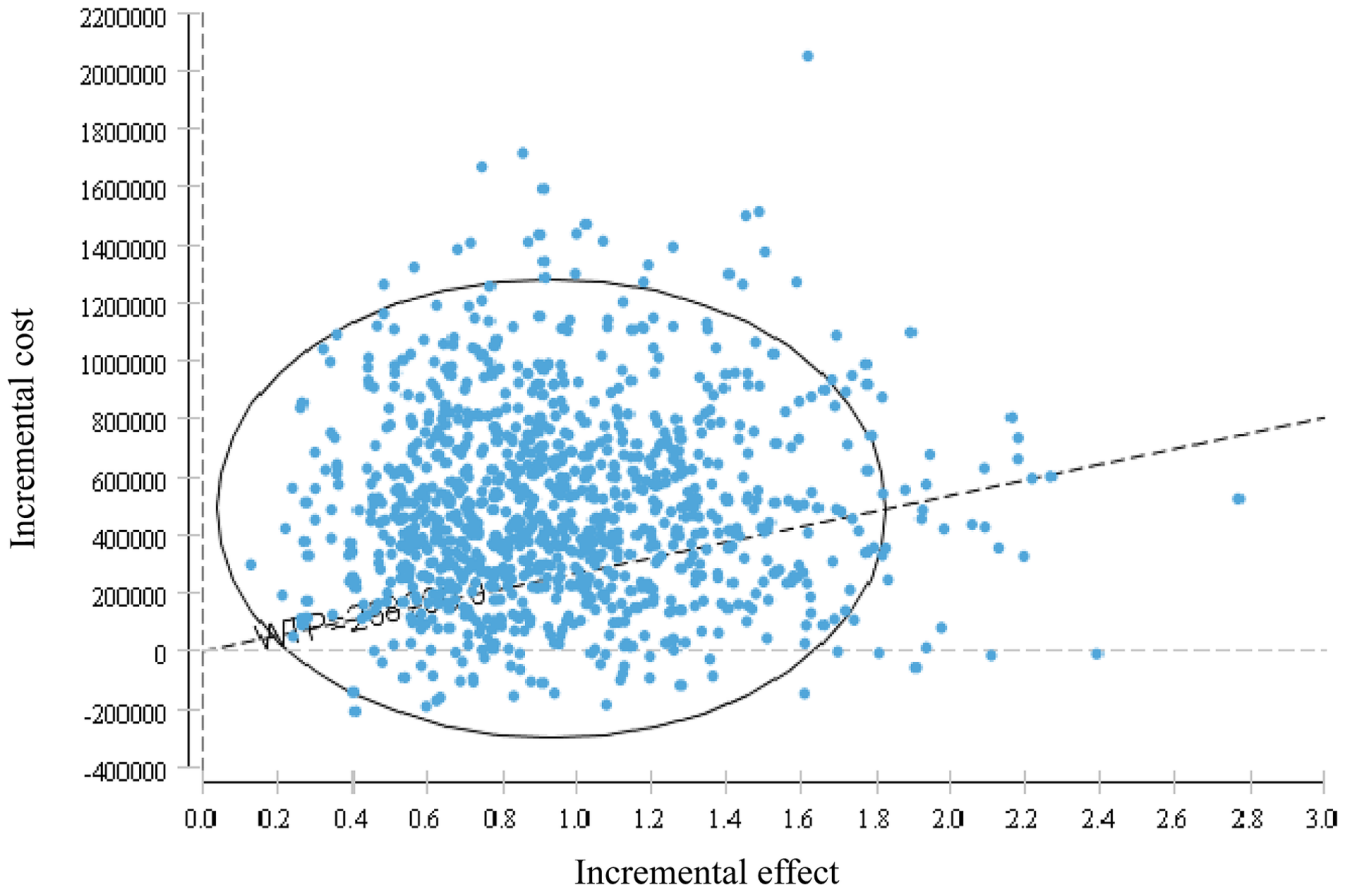
Incremental Cost-Effectiveness Scatterplot.

**Figure 8:**
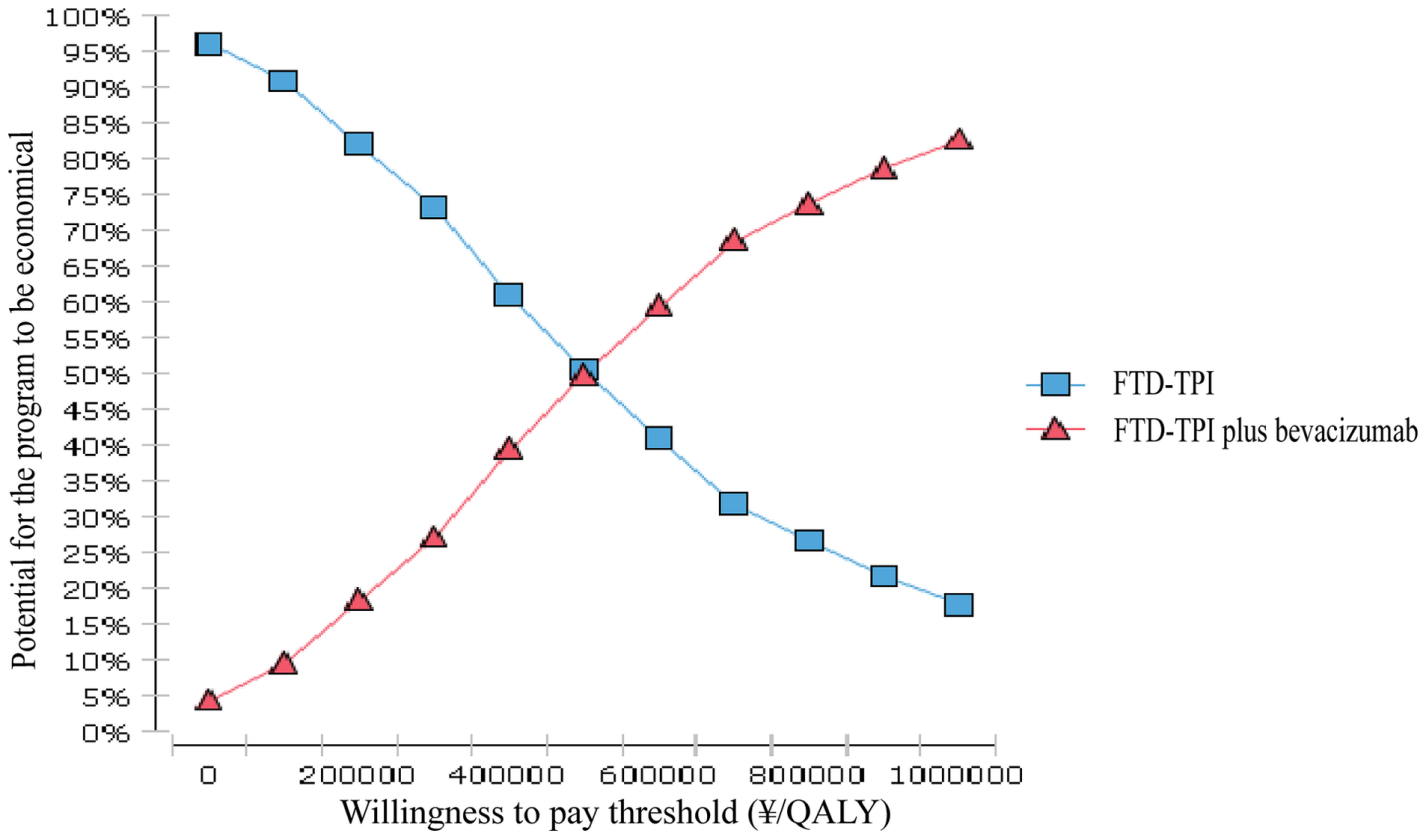
Cost-Effectiveness Acceptability Curve.

The probabilistic sensitivity analysis results are shown in Figures 7, 8. This study conducted 10,000 Monte Carlo simulations, revealing that as the incremental effect increases, the incremental cost also rises. Particularly at the willingness-to-pay threshold of ¥268,200.00, the majority of ICER values were above the threshold line, further corroborating the base case analysis conclusions. Figure 8 further illustrates that when the willingness-to-pay threshold is ¥494,000.00, the probability of the trifluridine/tipiracil combined with bevacizumab treatment regimen being cost-effective is 50.00%. As the willingness-to-pay threshold increases, the probability of cost-effectiveness also rises.

## 4 Discussion

Trifluridine/tipiracil is an oral combination of the nucleic acid analog trifluridine and the thymidine phosphorylase inhibitor tipiracil (7). Trifluridine acts as the active cytotoxic ingredient, forming trifluridine triphosphate when phosphorylated by thymidine kinase in tumor cells, which exerts its effects by substituting for thymidine in cellular DNA (7). Thymidine phosphorylase is responsible for the metabolism of trifluridine in the liver and gastrointestinal tract, converting it to an inactive form; however, the incorporation of tipiracil completely inhibits this degradation process, thereby significantly increasing the bioavailability of trifluridine.

The drug, which was introduced in the US and the EU in 2015 and 2016, respectively, and in China in 2019, is approved for use in patients with Metastatic Colorectal Cancer (mCRC) who have previously received fluoropyrimidine, oxaliplatin, and irinotecan-based chemotherapy. It is also indicated for mCRC patients who have either received or are not candidates for anti-vascular endothelial growth factor (VEGF) therapy, as well as for anti-epidermal growth factor receptor (EGFR) therapy in RAS wild-type mCRC patients. Bevacizumab primarily controls tumor growth by inhibiting neoangiogenesis (25) and is a key agent in the anti-angiogenic class of drugs for the treatment of advanced colorectal cancer.

Given the favorable efficacy and safety profile of trifluridine/tipiracil in the treatment of colorectal cancer, numerous studies have been conducted worldwide to explore its use in combination with targeted therapies. The Japanese C-TASK-FORCE study (26) demonstrated that in patients with advanced colorectal cancer and an Eastern Cooperative Oncology Group (ECOG) performance status (PS) of 0–1, the combination of trifluridine/tipiracil and bevacizumab as third-line therapy resulted in a centrally assessed 16-week PFS rate of 42.9% (80% CI, 27.8–59.0). The median PFS was 3.7 months (95% CI, 2.0–5.4), and the median OS was 11.4 months (95% CI, 7.6–13.9). The PFS exceeded the predefined threshold for trifluridine/tipiracil monotherapy. Additionally, the European DANISH study (27) showed that in patients with an ECOG PS of 0–1, the combination of trifluridine/tipiracil and bevacizumab was significantly more effective than monotherapy as third-line treatment. The median PFS was 2.6 months (95% CI, 1.6–3.5) in the trifluridine/tipiracil group, compared to 4.6 months (95% CI, 3.5–6.5) in the combination group (hazard ratio 0.45 [95% CI, 0.29–0.72]; *p* = 0.0015). In terms of safety, serious adverse events occurred in 21 patients (45%) in the trifluridine/tipiracil group and in 19 patients (41%) in the trifluridine/tipiracil plus bevacizumab group. These findings suggest that the combination of trifluridine/tipiracil and bevacizumab offers superior efficacy and safety compared to trifluridine/tipiracil monotherapy. Based on these clinical results, trifluridine/tipiracil with or without bevacizumab has been recommended as a standard third-line treatment for metastatic colorectal cancer in several authoritative guidelines, including the National Comprehensive Cancer Network (NCCN) Colon and Rectal Cancer Guidelines 2024.V1 (28), the CSCO Colorectal Cancer Guidelines 2023, and the European Society for Medical Oncology (ESMO) Guidelines for Metastatic Colorectal Cancer 2023. With the publication of the SUNLIGHT study results, the recommendation level for the trifluridine/tipiracil plus bevacizumab regimen has been further elevated in various national guidelines.

Colorectal cancer imposes a significant burden of disease and economic costs on patients in China and other regions worldwide. A study conducted in Saudi Arabia, which included 326 colorectal cancer patients, reported total healthcare costs of $19 million, with an average annual cost per patient of $58,384. Drug costs constituted the most significant proportion of healthcare expenditures, accounting for 45% of the total costs, followed by surgical costs (27%) (29). Another retrospective study (30) revealed that in 2020, the total healthcare costs for colorectal cancer in the United States reached $24.3 billion, representing 12.6% of all cancer treatment expenditures. The average cost per patient during the first year following diagnosis was $66,500, while the cost in the last 12 months of life escalated to $110,100. In Europe, the annual economic burden of colorectal cancer is estimated to be €19 billion, making it the most economically burdensome gastrointestinal cancer. Approximately half of this burden is attributed to direct healthcare costs, while the other half results from productivity losses, mortality, and caregiver opportunity costs. In 2022, it was estimated that China had 517,100 new cases of colorectal cancer and 240,000 related deaths, ranking it as the second leading cancer by incidence and the fourth by mortality among all malignancies (31). Colorectal cancer poses a particularly significant disease burden on the Chinese population compared to other cancers. In 2019, the DALYs attributable to colorectal cancer they reached 6,394,918 years, with an age-standardized DALY rate of 321.41 per 100,000, making it the third highest among all malignancies (32, 33). A retrospective study on the economic burden of clinical treatment for hospitalized colorectal cancer patients showed that 3.95% received third-line therapy. Western medicine costs comprise the most significant proportion of expenses (34). Given the current trends in colorectal cancer in China, it is crucial to evaluate the cost-effectiveness of trifluridine/tipiracil combined with bevacizumab compared to trifluridine/tipiracil monotherapy for third-line treatment of colorectal cancer.

In economic evaluations of colorectal cancer treatment, a study from Japan (35) assessed the cost-effectiveness of optimal treatment regimens for patients with KRAS wild-type metastatic colorectal cancer using FOLFIRI in combination with bevacizumab (Bmab), cetuximab (Cmab), or panitumumab (Pmab). The ICERs for Bmab, Cmab, and Pmab compared to FOLFIRI monotherapy were 736,700 JPY/month, 1,378,600 JPY/month, and 3,821,400 JPY/month, respectively. Although these combination therapies demonstrated superior clinical efficacy, none were considered cost-effective economically compared to FOLFIRI monotherapy. Additionally, a cost-effectiveness study in Indonesia indicated that combining FOLFOX or FOLFIRI with bevacizumab was not cost-effective for metastatic colorectal cancer (36). A study based on the U.S. healthcare system reported that adding bevacizumab to the FOLFOX regimen resulted in an ICER of 571,240 USD/QALY. In contrast, in second-line treatment, the ICER for combining bevacizumab with FOLFIRI was 364,083 USD/QALY (37). In a cost-effectiveness analysis focused on older adult Chinese patients with metastatic colorectal cancer, the ICER for combining capecitabine with bevacizumab was 95,564.33 USD/QALY compared to capecitabine monotherapy (38). These findings suggest that adding bevacizumab to chemotherapy regimens does not provide a cost-effectiveness advantage, which is consistent with the conclusions of this study.

A study based on the Japanese healthcare system, utilizing a sample of adults meeting the criteria of the C-TASK FORCE study and employing the Declining Exponential Approximation of Life Expectancy (DEALE) method to estimate transition probabilities, evaluated the cost-effectiveness of combining trifluridine/tipiracil with bevacizumab compared to trifluridine/tipiracil monotherapy. The study demonstrated that combining trifluridine/tipiracil and bevacizumab offered a cost-effectiveness advantage (39). However, this study was based on phase 1/2 clinical trial data, which had a small sample size and needed more reliable utility value data, limiting its generalizability. The study also highlighted the need for further evaluations based on clinical trial data to assess the cost-effectiveness of trifluridine/tipiracil combined with bevacizumab in colorectal cancer patients.

This study, from the perspective of the Chinese healthcare system, utilized the latest data from the SUNLIGHT study and applied parametric distribution fitting and Markov models to simulate the cost-effectiveness over 10 years. The results showed that the incremental effect of trifluridine/tipiracil combined with bevacizumab was 0.91 QALYs, with an ICER of ¥527,577.36/QALY, far exceeding the willingness-to-pay threshold of ¥268,200.00. Both one-way sensitivity analysis and probabilistic sensitivity analysis validated the robustness of the model. In the one-way sensitivity analysis, drug costs and the utility value of PFS had the greatest impact on the model’s output. According to these findings, the trifluridine/tipiracil combined with the bevacizumab treatment regimen does not offer a cost-effectiveness advantage.

The primary reason is the significant price difference between the two drug regimens. Additionally, the combined regimen resulted in more treatment-related adverse events, increasing the cost of managing these events. Although the combined regimen showed statistically significant improvements in PFS and OS rates, these benefits were offset by the additional treatment and management costs. Furthermore, the long-term advantages of the combined regimen in PFS and OS are expected to diminish over time. This study has several limitations. The utility values for various states of colorectal cancer patients in the Chinese population were primarily derived from previous similar studies, as there is currently a lack of authoritative utility values reflecting the different stages of the Chinese population. Therefore, the utility values may be biased. Additionally, this study extrapolated survival curves through parametric distribution fitting, but it was limited to the 12-month PFS and OS survival data provided by the SUNLIGHT study. Compared to the need for long-term survival data, the data changes may have significant errors. The survival probabilities obtained through parameter fitting may not fully reflect the actual state transitions of Chinese colorectal cancer patients. This study only included direct medical costs and did not account for indirect medical costs, which may lead to an underestimation of the total cost output by the model. We look forward to obtaining more clinical data and real-world data in the future to analyze further the cost-effectiveness of trifluridine/tipiracil combined with bevacizumab in treating colorectal cancer patients.

In conclusion, the current evidence suggests that trifluridine/tipiracil combined with bevacizumab does not have a cost-effectiveness advantage over trifluridine/tipiracil monotherapy in treating colorectal cancer patients.

## Data Availability

The original contributions presented in the study are included in the article/Supplementary material, further inquiries can be directed to the corresponding author.

## Footnotes

^1^ https://gpo.gzggzy.cn/

## Supplementary material

SUPPLEMENTARY FIGURE S1 Parameters of OS curve fitting for FTP-TPI.

SUPPLEMENTARY FIGURE S2 Parameter plot of PFS curve fitting for FTP-TPI.
