# Supplementary figures and images for: Cost-effectiveness analysis of trifluridine/tipiracil combined with bevacizumab vs. monotherapy for third-line treatment of colorectal cancer
Source: Front Public Health. 2024 Nov 13;12:1465898. doi: 10.3389/fpubh.2024.1465898 (PMC11599266; doi:10.3389/fpubh.2024.1465898)

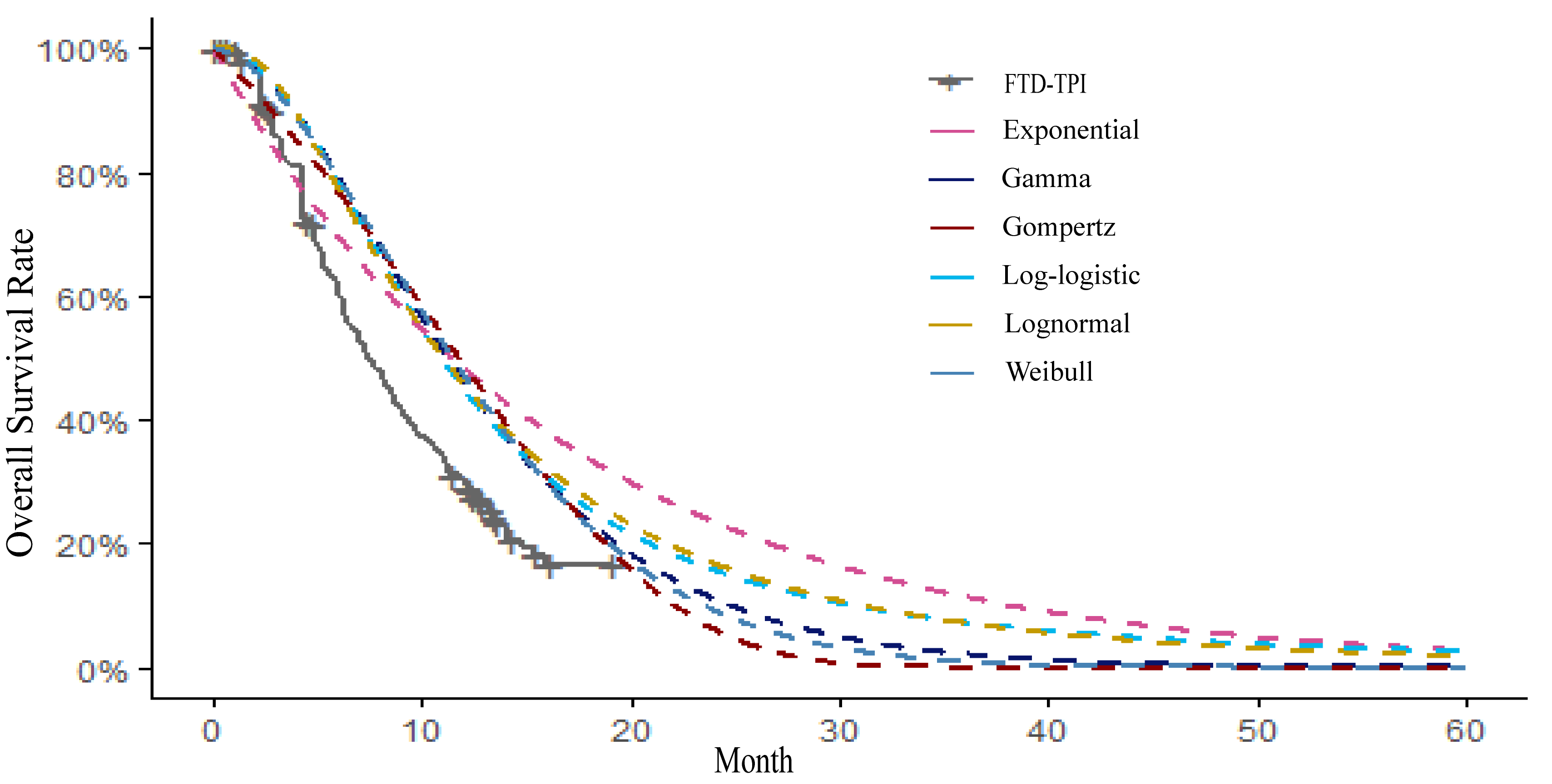

Supplement: SUPPLEMENTARY FIGURE S1 — Parameters of OS curve fitting for FTP-TPI. [file Image_1.tif]

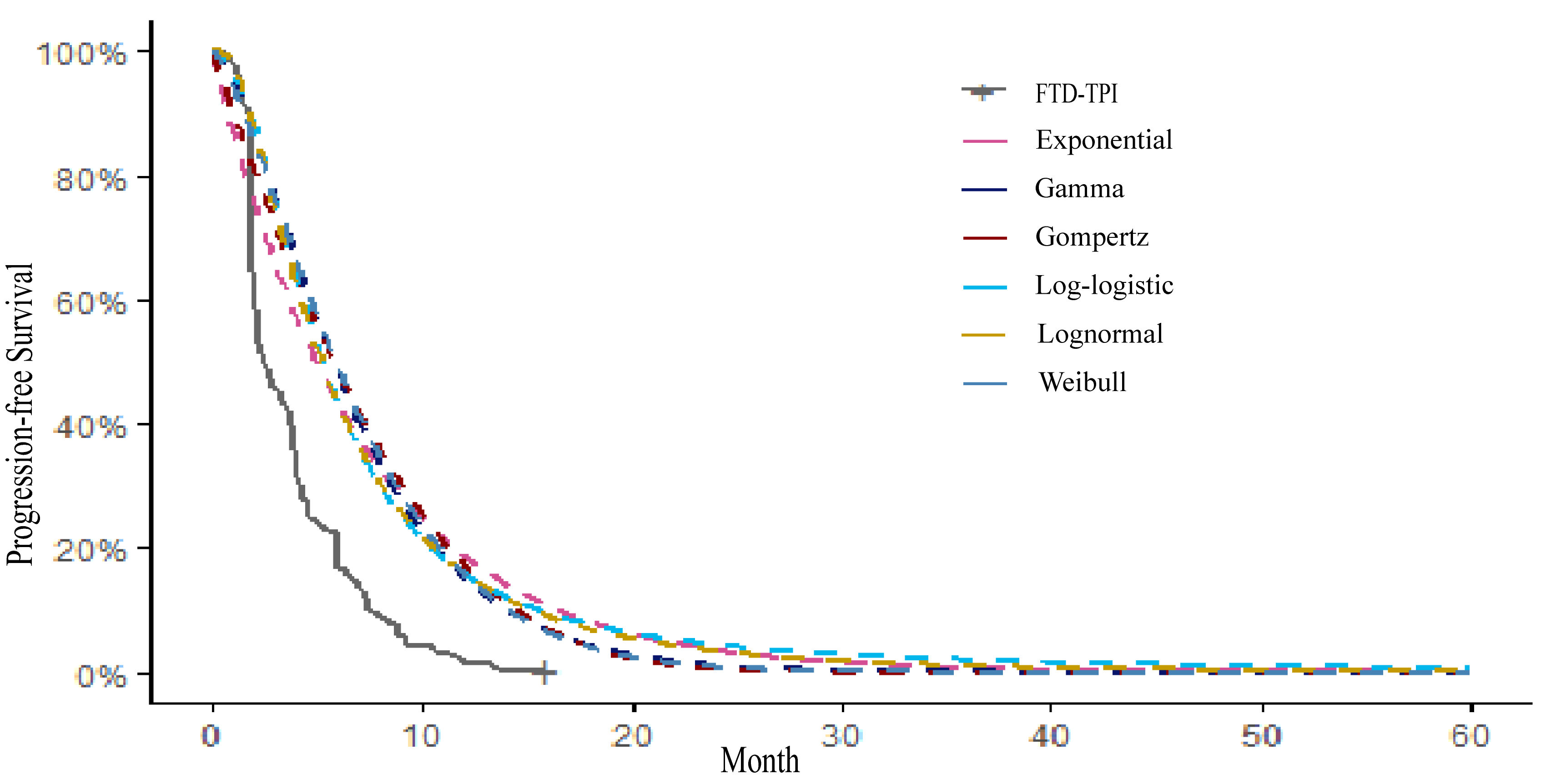

Supplement: SUPPLEMENTARY FIGURE S2 — Parameter plot of PFS curve fitting for FTP-TPI. [file Image_2.tif]
