# Supplementary material for: Cost-effectiveness analysis of trifluridine/tipiracil combined with bevacizumab vs. monotherapy for third-line treatment of colorectal cancer
Source: Front Public Health. 2024 Nov 13;12:1465898. doi: 10.3389/fpubh.2024.1465898 (PMC11599266; doi:10.3389/fpubh.2024.1465898)
Supplement: Supplementary file 3 [file Table_1.docx]

| Table S1 Results of AIC and BIC for Model Fitting of Various Parameters | | | | | |
| --- | --- | --- | --- | --- | --- |
| Group | Distribution Type | OS Fitted AIC Value | OS Fitted BIC Value | PFS Fitted AIC Value | PFS Fitted BIC Value |
| FTP-TPI plus bevacizumab | Exponential | 1 082.722 | 1 086.227 | 1 197.485 | 1 200.990 |
|  | Weibull | 1 042.854 | 1 049.865 | 1 168.387 | 1 175.398 |
|  | Log-logistic | 1 033.415 | 1 040.426 | 1 159.565 | 1 166.576 |
|  | Log-normal | 1 031.027 | 1 038.038 | 1 153.674 | 1 160.685 |
|  | Gompertz | 1 062.891 | 1 069.902 | 1 185.819 | 1 192.829 |
|  | Gamma | 1 036.950 | 1 043.961 | 1 341.929 | 1 348.940 |
|  | Distribution Type | OS Fitted AIC Value | OS Fitted BIC Value | PFS Fitted AIC Value | PFS Fitted BIC Value |
| FTP-TPI | Exponential | 1 148.323 | 1 151.828 | 1 069.348 | 1 072.854 |
|  | Weibull | 1 110.076 | 1 117.086 | 1 009.450 | 1 016.461 |
|  | Log-logistic | 1 097.71 | 1 104.721 | 964.470 | 971.480 |
|  | Log-normal | 1 092.397 | 1 099.408 | 955.090 | 962.100 |
|  | Gompertz | 1 130.98 | 1 137.99 | 1 051.143 | 1 058.154 |
|  | Gamma | 1 110.810 | 1 117.820 | 1 061.658 | 1 068.669 |
